# The first genome sequence of a metatherian herpesvirus: Macropodid herpesvirus 1

**DOI:** 10.1186/s12864-016-2390-2

**Published:** 2016-01-22

**Authors:** Paola K. Vaz, Timothy J. Mahony, Carol A. Hartley, Elizabeth V. Fowler, Nino Ficorilli, Sang W. Lee, James R. Gilkerson, Glenn F. Browning, Joanne M. Devlin

**Affiliations:** Asia Pacific Centre for Animal Health, Faculty of Veterinary and Agricultural Sciences, The University of Melbourne, Building 400, Parkville, 3010 VIC Australia; Queensland Alliance for Agriculture and Food Innovation, Centre for Animal Science, The University of Queensland, Ritchie Building (64A), St Lucia, 4072 QLD Australia; Department of Agriculture and Fisheries, Animal Science, St Lucia, 4072 QLD Australia; College of Veterinary Medicine, Konkuk University, Seoul, 143-701 Republic of Korea

**Keywords:** Marsupial, Herpesvirus, Genome, Wildlife, Macropod, Kangaroo, Herpes simplex

## Abstract

**Background:**

While many placental herpesvirus genomes have been fully sequenced, the complete genome of a marsupial herpesvirus has not been described. Here we present the first genome sequence of a metatherian herpesvirus, *Macropodid herpesvirus 1* (MaHV-1).

**Results:**

The MaHV-1 viral genome was sequenced using an Illumina MiSeq sequencer, *de novo* assembly was performed and the genome was annotated. The MaHV-1 genome was 140 kbp in length and clustered phylogenetically with the primate simplexviruses, sharing 67 % nucleotide sequence identity with *Human herpesviruses 1* and *2*. The MaHV-1 genome contained 66 predicted open reading frames (ORFs) homologous to those in other herpesvirus genomes, but lacked homologues of UL3, UL4, UL56 and glycoprotein J. This is the first alphaherpesvirus genome that has been found to lack the UL3 and UL4 homologues. We identified six novel ORFs and confirmed their transcription by RT-PCR.

**Conclusions:**

This is the first genome sequence of a herpesvirus that infects metatherians, a taxonomically unique mammalian clade. Members of the *Simplexvirus* genus are remarkably conserved, so the absence of ORFs otherwise retained in eutherian and avian alphaherpesviruses contributes to our understanding of the *Alphaherpesvirinae.* Further study of metatherian herpesvirus genetics and pathogenesis provides a unique approach to understanding herpesvirus-mammalian interactions.

**Electronic supplementary material:**

The online version of this article (doi:10.1186/s12864-016-2390-2) contains supplementary material, which is available to authorized users.

## Background

Members of the *Herpesviridae* have a linear double-stranded DNA genome, 120–245 kbp in length, and cause significant morbidity and mortality in diverse groups of animals. Members are further classified into three subfamilies; the *Alpha*-, *Beta*- and *Gammaherpesvirinae*. Reports of herpesvirus infections in the *Marsupialia* date back to the 1970s. The first isolation of a marsupial herpesvirus was from a fatal outbreak of severe respiratory disease and systemic organ failure in a zoological collection of Parma wallabies (*Macropus parma*) [1]. The isolation of this alphaherpesvirus, designated *Macropodid herpesvirus 1* (MaHV-1), was closely followed by the isolation of a second, related, herpesvirus (*Macropodid herpesvirus 2*, MaHV-2) from fatal cases of disease in several vulnerable macropod species [2]. The macropodid viruses were detected in animals displaying some clinical signs of disease similar to those caused by *Human herpesviruses 1* and *2* (HHV-1 and −2) infection, such as conjunctivitis and vesicular anogenital lesions, but also included hepatic disease [1, 3].

In the following 30 years, before the identification of additional marsupial herpesviruses, evidence of herpesvirus infection in metatherians was largely observed through electron microscopy or sero-epidemiological surveys. These sero-prevalence studies measured neutralising antibodies to MaHV-1, which were detected in both wild (23–69 %) and captive (41 %) populations of marsupials [4, 5]. Since 2008, eleven additional marsupial herpesviruses have been identified, including a closely-related alphaherpesvirus, *Macropodid herpesvirus 4* (MaHV-4), in free-ranging eastern grey kangaroos (*Macropus giganteus*) with clinical signs of respiratory and possible neurological disease [6] and two gammaherpesviruses from macropods [7–9]. A further eight herpesviruses have been identified in other (non-macropodid) marsupial species, though little sequence data are available for these viruses [9–13].

Despite its classification as a *Simplexvirus*, early genome hybridization studies of MaHV-1 identified a type D genome structure (as defined by [14]) of approximately 135 kbp in length, containing a short unique (U_S_) region, flanked by large inverted repeat sequences (internal repeat and terminal repeat; IR_S_/TR_S_) joined to a long unique (U_L_) region [15, 16]. MaHV-1 occurs as only two equimolar genomic isomers [15]. These genomic features are characteristic of *Varicelloviruses* such as varicella zoster virus (VZV) and pseudorabies virus (PRV) and contrast with those of MaHV-2. MaHV-2 has a type E genome arrangement, more typical of the *Simplexviruses*, and occurs as four equimolar genomic isomers [17]. To date MaHV-1 is the only alphaherpesvirus that encodes both ICP34.5 (RL1) and the host-derived oncogene thymidylate synthase [18]. Sequence analysis of two conserved ORFs in MaHV-1, −2 and −4, as well as analyses of their antigenic relationships, has clustered these macropodid viruses closely with the primate simplexviruses [3, 6, 19]. As metatherian and eutherian mammals are believed to have diverged over 85 million years ago [20], this viral phylogenetic grouping differed from the typical virus-host co-evolutionary pattern observed within the *Herpesviridae* [3, 19, 21, 22] and was instead suggestive of a recent and complex speciation event.

This study aimed to sequence and analyse the full genome of the metatherian alphaherpesvirus, MaHV-1, with particular attention to novel genomic features.

## Results and discussion

### Whole genome sequence analysis

**Fig. 1:**
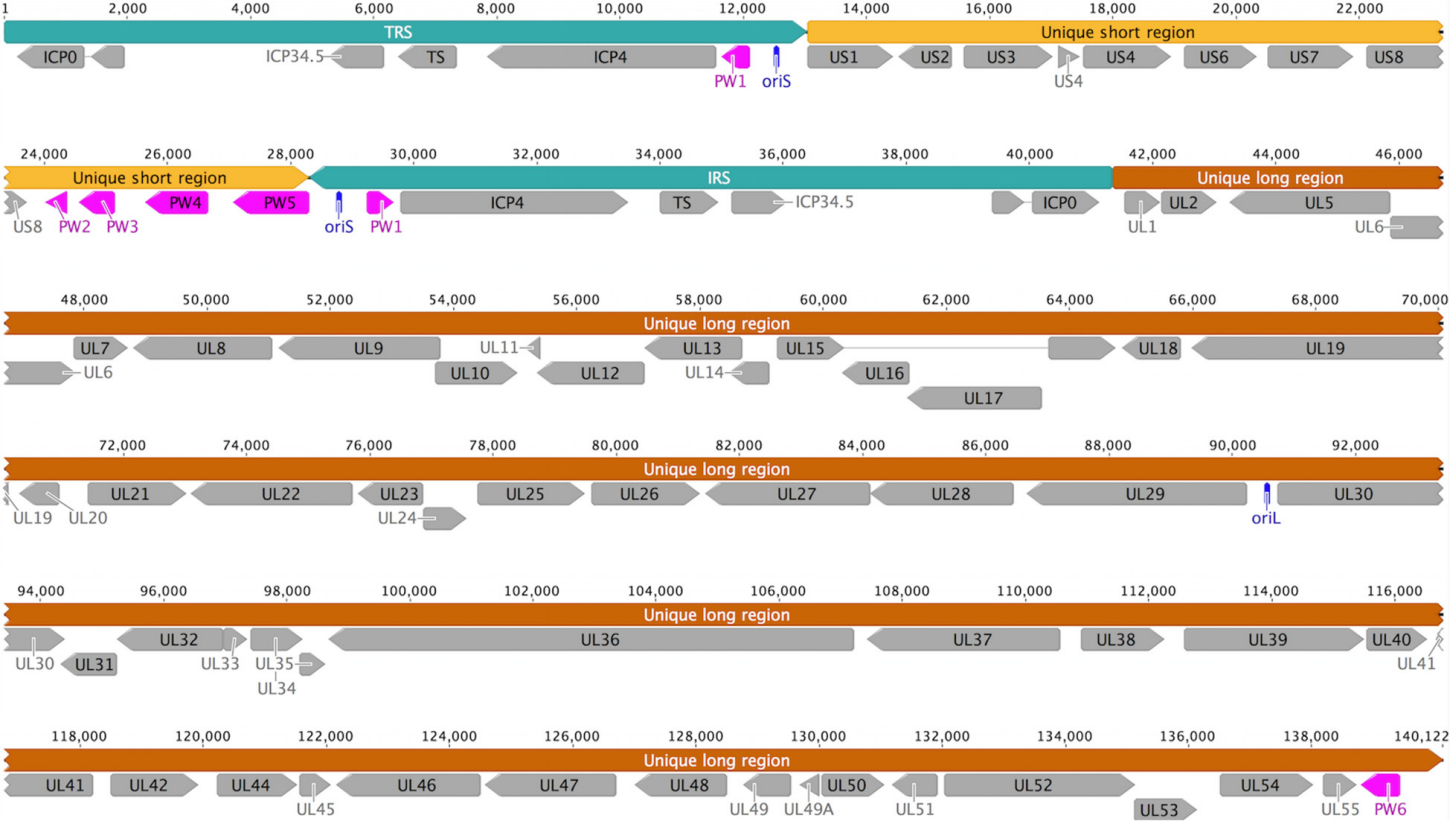
Schematic representation of the genome arrangement of *Macropodid herpesvirus 1*. Herpesviral conserved ORFs are annotated in grey using the HHV-1 and HHV-2 ORF nomenclature. Novel ORFs (*pink*) are given a PW prefix. Unique long genome region (*orange*), Unique short genome region (*yellow*), TRS/IRS = inverted repeat flanking unique short region (terminal and internal, light blue), oriL/S = origins of replication (*dark blue*)

The genome of MaHV-1 is the first metatherian herpesvirus to be sequenced. Excluding the genomic termini, which remained unresolved, the final genome length of MaHV-1 was approximately 140.1 kbp (Fig. 1) [GenBank:KT594769], larger than previously predicted. This difference appears to be due to a larger than predicted inverted repeat region [15]. This included a 98.8 kbp U_L_ region and a 15.3 kbp U_S_ region flanked by 13 kbp inverted repeat sequences (IR_S_/TR_S_). The MaHV-1 genome had a G + C content of 52.9 %, but had a higher G + C content (up to 61.7 %) within the IR_S_/TR_S_ regions. The final genome assembly had a mean depth of 2,168 reads per bp (2.05 million mapped reads) and approximately 95 % of reads had a quality score of at least Phred_20_. Three origins of replication were identified. The origin of lytic replication (oriLyt) was located between UL29 and UL30 in the U_L_ region and the oriS was located within the IRs/TRs regions. Thus two copies of oriS were present, as in the genomes of HHV-1 and −2.

### Conserved alphaherpesvirus ORFs

**Fig. 2:**
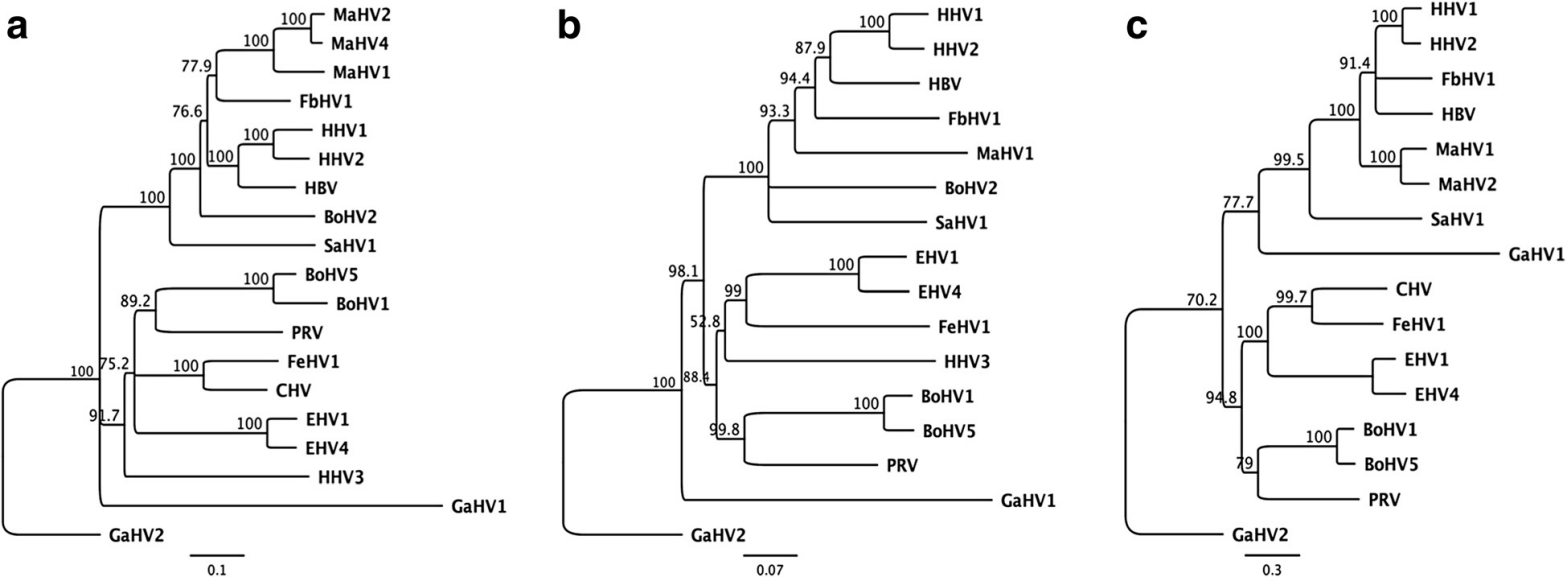
The relationship between macropodid herpesvirus 1 (MaHV1) and other viruses from the *Alphaherpesvirinae* subfamily. Neighbour-joining distance trees were generated using the translated protein sequences of conserved herpesvirus ORFs: **a** glycoprotein B, gB (UL27); **b** DNA polymerase, DPOL (UL30); and **c** glycoprotein D, gD (US6). Bootstrap values (10,000 replicates) are shown for each branch, with Gallid herpesvirus 2 (GaHV2) as an outgroup. The abbreviations and GenBank accession details are: macropodid herpesvirus 2 (MaHV2) [GenBank:AAD11961, GenBank:AAL13143, gB and gD], macropodid herpesvirus 4 (MaHV4) [GenBank:AGC54689, gB], macacine herpesvirus 1 (HBV) [GenBank:AAA85652, GenBank:NP_851890, GenBank:AAB24129, gB, DPOL and gD], fruit bat herpesvirus 1 (FbHV1) [GenBank:YP_009042089, GenBank:YP_009042092, GenBank:YP_009042126, gB, DPOL and gD], gallid herpesvirus 1 (GaHV1) [GenBank:AEB97319 , GenBank:AEB97322, GenBank:AEB97368, gB, DPOL and gD], gallid herpesvirus 2 (GaHV2) [GenBank:CAA63039, GenBank:YP_001033959, GenBank:AAA64967, gB, DPOL and gD], human herpesvirus 1 (HHV1) [GenBank:NP_044629, GenBank:NP_044632, GenBank:CAA32283, gB, DPOL and gD], human herpesvirus 2 (HHV2) [GenBank:NP_044497, GenBank:NP_044500, GenBank:AAB60553, gB, DPOL and gD], human herpesvirus 3 (HHV3) [GenBank:NP_040154.2, GenBank:NP_040151, gB and DPOL], saimiriine herpesvirus 1 (SaHV1) [GenBank:YP_003933812, GenBank:YP_003933809, GenBank:ADO13836, gB, DPOL and gD], bovine herpesvirus 1 (BoHV1) [GenBank:AAA46013, GenBank:NP_045328, GenBank:CAA80604, gB, DPOL and gD], bovine herpesvirus 2 (BoHV2) [GenBank:P12641.2, GenBank:AAD55134:, gB and DPOL], bovine herpesvirus 5 (BoHV5) [GenBank:YP_003662497, GenBank:YP_003662494, GenBank:AAA67359, gB, DPOL and gD], canine herpesvirus 1 (CHV) [GenBank:AAK51052, GenBank:AAX47050, GenBank:AAB67058, gB, DPOL and gD], felid herpesvirus 1 (FeHV1) [GenBank:AAB28559, GenBank:YP_003331549, GenBank:BAA06442, gB, DPOL and gD], equine herpesvirus 1 (EHV1) [GenBank:Q6DLH8, GenBank:YP_053075, GenBank:AAA46073, gB, DPOL and gD], equine herpesvirus 4 (EHV4) [GenBank:NP_045250, GenBank:NP_045247, GenBank:NP_045289, gB, DPOL and gD], suid herpesvirus 1 (PRV) [GenBank:YP_068330, GenBank:YP_068333, GenBank:AAC36717, gB, DPOL and gD]

The U_L_ region of the MaHV-1 genome encoded 54 ORFs common to other herpesviruses (Table 1). The predicted protein sequences of these ORFs shared between 41 % and 73 % aa pairwise identity (up to 86 % aa similarity) with HHV-1 and −2 homologues. In the U_S_ region the MaHV-1 genome encoded seven ORFs common to other simplexviruses (US1 to US4 and US6 to US8), with the predicted protein sequences sharing between 32 and 59 % aa pairwise identity (up to 73 % aa similarity) with HHV-1 and −2 homologues. The IR_S_/TR_S_ regions encoded five ORFs, including those for thymidylate synthase, ICP0 and ICP34.5. There were no homologues of UL3, UL4, UL56 or US5 (glycoprotein J, gJ) identified in the MaHV-1 genome. Also, the US4 (glycoprotein G, gG) homologue was predicted to be non-functional, as the ORF was prematurely truncated (120 aa residues compared to 583 aa in MaHV-4). This is consistent with previous published sequence data reporting a truncation of the MaHV-1 gG ORF [6, 23]. Phylogenetic analyses using translated protein sequences of three core herpesvirus genes (UL27, UL30 and US6) are shown in Fig. 2. These analyses show that MaHV-1 clusters most closely with other macropodid herpesviruses (MaHV-2 and MaHV-4), as well as with the simplexviruses that infect primates. It also groups with the herpesvirus of an Indonesian pteropodid bat. Comparison of other viral core genes yielded similar clustering patterns. Comparison of the MaHV-1 UL27 and UL30 ORFs with those of the recently sequenced fruit bat herpesvirus 1 (FbHV-1) [GenBank:BAP00706 and GenBank:YP_009042092; UL27 and UL30, respectively] showed that these ORFs shared 71 and 67 % pairwise aa identity, respectively (83 and 78 % aa similarity). This similarity is comparable to that seen between MaHV-1 and HHV-1/HHV-2 (Table 1 and Fig. 2), which may offer some insight into their evolutionary relationship, for example, may suggest transmission of herpesviruses from primates to bats, and then to marsupials. Sequencing of herpesviruses from other metatherians, as well as other Australasian mammals, will be needed to determine the significance of this clustering.

**Table 1:** Predicted open reading frames (ORFs) identified in different structural regions of the *Macropodid herpesvirus 1* genome and percentage pairwise amino acid identity to ORFs in related alphaherpesviruses

				% aa *pairwise* identity^#^			
ORF	Length (nt)	Length (aa)	Description (putative) ^§^	MaHV2	MaHV4	HHV1	HHV2
Unique short region							
US1	1392	463	Infected cell protein 22; ICP22			29	35
US2	867	288	Tegument protein			49	49
US3	1449	482	Protein kinase			56	59
US4	360	119	Glycoprotein G; predicted non-functional	59	63	32	35
US6	1188	395	Glycoprotein D	74		56	57
US7	1392	463	Glycoprotein I	58		38	39
US8	1599	532	Glycoprotein E	63		34	32
PW2	342	113	Unique hypothetical				
PW3	582	193	Unique hypothetical				
PW4	1023	340	Unique hypothetical				
PW5	1233	410	Unique hypothetical				
Inverted repeat region							
PW1	453	150	Unique hypothetical				
ICP4	3708	1235	Infected cell protein 4; ICP4			51	50
TS	951	316	Thymidylate synthase; host-derived				
ICP34.5	900	299	Infected cell protein 34.5; ICP34.5			62	56
ICP0	1629	542	Infected cell protein 0; ICP0			50	51
Unique long region							
UL1	579	192	Glycoprotein L			41	47
UL2	906	301	Uracil-DNA glycosylase			59	60
UL5	2601	866	Helicase-primase helicase subunit			73	73
UL6	2007	668	Capsid portal protein			62	63
UL7	888	295	Tegument protein			51	49
UL8	2253	750	Helicase-primase subunit			38	39
UL9	2616	871	DNA replication origin-binding helicase			70	70
UL10	1347	448	Glycoprotein M			51	48
UL11	246	81	Myristoylated tegument protein			45	59
UL12	1749	582	Alkaline exonuclease; deoxyribonuclease			56	56
UL13	1845	614	Tegument serine/threonine protein kinase			48	47
UL14	645	214	Tegument protein			60	63
UL15	2100	699	DNA packaging terminase subunit 1			70	71
UL16	1098	365	Tegument protein			51	54
UL17	2184	727	DNA packaging tegument protein			51	50
UL18	951	316	Capsid triplex subunit 2			68	68
UL19	4131	1376	Major capsid protein			69	70
UL20	648	215	Envelope protein			45	43
UL21	1596	531	Tegument protein			43	43
UL22	2631	876	Glycoprotein H			45	45
UL23	1062	353	Thymidine kinase	76		42	44
UL24	702	233	Nuclear protein			52	53
UL25	1740	579	DNA packaging tegument protein			61	61
UL26	1761	586	Capsid maturation protease			53	52
UL27	2664	887	Glycoprotein B	83	82	72	70
UL28	2325	774	DNA packaging terminase subunit 2			64	63
UL29	3579	1192	ICP8; single stranded binding protein			70	69
UL30	3675	1224	DNA polymerase			66	66
UL31	921	306	Nuclear egress lamina protein			73	72
UL32	1731	576	DNA packaging protein			62	61
UL33	390	129	DNA packaging protein			61	60
UL34	837	278	Nuclear egress membrane protein			44	43
UL35	408	135	Small capsid protein			53	50
UL36	8535	2844	Large tegument protein			48	48
UL37	3150	1049	Tegument protein			46	45
UL38	1359	452	VP19C; capsid triplex subunit 1			56	56
UL39	2916	971	Ribonucleotide reductase subunit 1			65	66
UL40	990	329	Ribonucleotide reductase subunit 2			65	65
UL41	1566	521	Tegument host shutoff protein			60	61
UL42	1422	473	DNA polymerase processivity subunit			44	44
UL44	1296	431	Glycoprotein C			36	34
UL45	516	171	Membrane protein			42	40
UL46	2343	780	VP11/12; tegument protein			49	49
UL47	2133	710	VP13/14; tegument protein			43	42
UL48	1497	498	VP16; transactivating tegument protein			47	47
UL49	777	258	VP22; tegument protein			67	67
UL49A	324	107	Glycoprotein N				32
UL50	1035	344	Deoxyuridine triphosphatase			39	35
UL51	741	246	Tegument protein			67	66
UL52	3102	1033	Helicase primase subunit			59	58
UL53	1023	340	Glycoprotein K			46	47
UL54	1524	507	ICP27; multifunctional expression regulator			52	52
UL55	540	179	Nuclear protein			40	36
PW6	630	209	Unique hypothetical				

^§^Putative function of encoded polypeptides in MaHV-1

^#^amino acid identities with homologues in *Human herpesvirus 1* and *2* (HHV1 and HHV2) and *Macropodid herpesviruses* 2 and 4 (MaHV2 and MaHV4)

Although UL3 and UL4 are conserved in all other alphaherpesviruses examined to date, gene deletion studies in the human simplexviruses have found that deletion of UL3 and UL4 does not affect viral replication or cell-to-cell spread in vitro [24]. In vivo functions of the UL3 and UL4 encoded accessory proteins are not well understood, but they colocalise and directly interact with the transcriptional repressor ICP22, encoded by US1, in small dense nuclear bodies and may also be involved in the late phase of viral replication [25–27]. The absence of gJ is also of note. This is the third *Simplexvirus* found to lack an ORF encoding a gJ homologue, which is otherwise conserved in the *Simplexviru*s genus. The other two simplexviruses lacking gJ are leoporid herpesvirus 4 and FbHV-1 [28, 29]. In other herpesviruses gJ inhibits host cell apoptosis by inducing an increase in concentrations of reactive oxygen species in the host cell [30]. It is unclear whether the absence of UL3, UL4 and gJ might be related to adaptation to a new host (marsupials) or whether it may be the result of virus passage in vitro. In respect to gJ, the former scenario could be more likely, as the absence of this ORF in other non-primate herpesviruses shows that it is not strictly conserved within the genus. Sequencing of other marsupial alphaherpesviruses, particularly field isolates, would help to resolve this finding.

### Unique or hypothetical ORFs

Seven unique hypothetical ORFs were identified; one in the U_L_ region, two located in the IR_S_/TR_S_ regions, and four in the U_S_ region. Viral transcript analyses by qRT-PCR confirmed that six of the seven predicted ORFs were transcribed at both 4 and 12 h post infection (hpi) under in vitro conditions (Additional file 1: Figure S1). No transcripts for these six ORFs were detected in the uninfected cell controls at any time point. The seventh predicted ORF, which was located in the large inverted repeat region flanked by ICP0 and ICP34.5, was excluded from further analyses as qRT-PCR targeting this ORF could not confirm transcription*.* The six ORFs for which transcription was confirmed were annotated PW1 to PW6. PW1 was encoded in the TR_S_/IR_S_ repeat region (and thus two copies were present), and no significant structural or sequence domains or motifs were identified within it. Four novel ORFs, PW2 to PW5, were encoded in the U_S_ region as a cluster downstream of US8 (Fig. 1). No putative conserved domains were detected in the polypeptides encoded by PW2 and 4, although PW3 and 5 had increased internal hydrophobicities, suggestive of transmembrane domains. Structural prediction analyses using I-TASSER suggested potential structural homologues for PW3 (*C. elegans* SMG5-7 complex for nonsense-mediated mRNA decay, [PDBHit:3zheB2], TM-score = 0.805, RMSDa = 2.69, 94.3 % coverage) and PW4 (Phage Phi6 capsid subunit, [PDBHit:4btgA], TM-score = 0.797, RMSDa = 2.64, 87.6 % coverage). No significant structural homologues were predicted for any of the other novel ORFs. Analysis of the predicted amino acid sequence of PW5 detected a microneme/rhoptry antigen domain [PSSM ID:185628]. There was only one novel predicted ORF within the U_L_ region, PW6. This ORF was identified downstream of UL55. Analysis of the sequence of PW6 did not detect any conserved functional domain or motifs, but a hydrophobic domain near the carboxyl terminus was identified, suggestive of a transmembrane domain.

The MaHV-1 genome lacked an identifiable UL56 homologue. Studies in HHV-2 have shown that UL56 encodes a tegument protein involved in relocalising ubiquitin ligase Nedd4 in HHV-2 infected cells, and has a role in intracellular virion transport and/or virion release from the cell surface [31, 32]. UL56 polypeptide interacts and complexes with UL11 polypeptide as they co-localise in the Golgi apparatus and in aggresome-like structures [33]. In HHV-2, UL56 is dispensable for virus growth in vitro, but deletion of it results in reduced production of cell-free infectious virus [31]. In vivo, the presence of UL56 is important for pathogenicity of HHV-1, with deletion mutants having reduced neuroinvasiveness [34]. The hydrophobic C-terminal region of UL56 is particularly important for pathogenicity [35]. A similar hydrophobic region was identified in the C-terminus of PW6, which was encoded directly downstream of UL55. This may indicate that PW6 is a distant UL56 homologue, although it only shared 17 % aa pairwise identity (27 % aa similarity) with HHV-1 UL56. However, at this stage any structural or functional similarities between PW6 and UL56 remain unclear, particularly as preliminary analyses of predicted tertiary structures did not identify significant structural similarities.

In the absence of conserved motifs or domains, the sequences of PW1 to PW4 provide no indication of the potential functions of these novel polypeptides. The identification of a rhoptery antigen domain in PW5 may suggest an association with organelles, but little else can be inferred. High relative levels of transcript of PW2 to PW5 at 4 hpi may indicate that they are transcribed at an early stage of the replication cycle, but further studies are necessary to better differentiate the kinetics of expression of these ORFs. It is not clear whether these genes are important for in vitro replication or in vivo pathogenicity. Gene deletion studies or functional studies of the products of these ORFs would be necessary to elucidate their function. The clustering of novel ORFs identified in the US/IR region, PW1 to PW5, suggest that they may have been acquired in a single event, possibly from an unknown host or another virus during virus speciation. Sequence comparisons with other marsupial herpesviruses would help determine whether the novel ORFs are unique to MaHV-1, or are instead ORFs common to herpesviruses infecting metatherians.

## Conclusions

This is the first genome sequence of a herpesvirus that infects metatherians, a taxonomically unique mammalian clade. Members of the *Simplexvirus* genus are remarkably conserved, so the absence of ORFs otherwise conserved in eutherian and avian alphaherpesviruses contributes to our understanding of the *Alphaherpesvirinae* more generally. Together with the sequence similarities observed to the human herpesviruses, these conclusions indicate that further study of metatherian herpesvirus genetics and pathogenesis will provide a unique approach to understanding herpesvirus-mammalian interactions.

## Methods

### Viral genome sequencing and analysis

The MaHV-1 isolate selected for sequencing (MaHV1.3076/08) was originally isolated from a Parma wallaby with clinical signs of disease [1]. The viral nucleocapsid genomic DNA was purified and sequenced as previously described [6, 36]. Briefly, 50 ng of viral genomic DNA was used to prepare libraries using the Illumina Nextera DNA library preparation kits according to the manufacturer’s instructions. The libraries were pooled in equimolar concentrations and loaded onto an Illumina MiSeq. Sequencing was carried out using a 300 cycle V2 SBS kit (Illumina, Inc.) in paired-end 150 bp format. Over 350 Mbp of sequence data were obtained from 2.69 million paired reads with a mean length of 137 bp (standard deviation of 26.3) and were submitted to the Short Read Archive [SRA:SRP067309]. Reads were trimmed to an error probability limit of 0.5 % and de novo assembly was performed using medium-low default sensitivity settings on the bioinformatics package Geneious version 6.1.7 [37] (Biomatters Ltd). This yielded four large contigs (52.6 kbp, 37.3 kbp, 14.9 kbp and 17 kbp) with consensus sequences that corresponded to herpesvirus sequence, according to Blastx and Blastn searches of GenBank databases [38, 39]. These consensus sequences were used as references in further assemblies, where reads were reiteratively mapped until there was no further contig extension. Previously published MaHV-1 genome sequence data [GenBank:AY048539, GenBank:AF188480] was used to aid scaffold construction. Medium and high sensitivity default settings with a minimum of 90–95 % overlap identity in Geneious version 6.1.7 were used in these assemblies.

Prediction of open reading frames (ORFs) using Glimmer3 was restricted to those larger than 240 bp, and ORF annotations were determined by Blastx and Blastn searching against the NCBI non-redundant protein and nucleotide databases, respectively [38, 39]. ORF annotations followed those of HHV-1 and −2, whilst the novel ORFs were prefixed with PW (Parma wallaby). The unique MaHV-1 ORF sequences were translated to hypothetical polypeptides and compared to sequence motifs in the Pfam database to determine their putative functions. Further structural prediction analyses were performed using I-TASSER [40]. Threshold cut-off values of >1 for the normalised Z-score, < 3.0 for the RMSD and >0.7 for the TM-score were considered significant and used to identify structural homologues.

Phylogenetic analyses of the translated protein sequences of the core herpesvirus genes UL27, UL30 and US6 were performed using the neighbour - joining method in Geneious version 6.1.7 with the Jukes Cantor model of amino acid substitution [41]. Ten thousand bootstrap replicates were used to assess the significance of the phylogenetic tree topology.

### Confirmation of transcription of novel ORFs

To determine if the novel ORFs were transcribed in vitro, RNA from infected cells was interrogated using quantitative reverse transcription PCR (qRT-PCR). One-step growth analyses using wallaby fibroblast JU56 cells [42] in 6-well trays was performed as previously described [6] with modifications. Briefly, JU56 cells were infected with virus at a multiplicity of infection of 3 (3 median tissue culture infective dose (TCID_50_) per cell). The contents of wells collected at 4 and 12 hpi. RNA was extracted using the RNeasy RNA Extraction kit (Qiagen) and 2 μg of purified nucleic acid was treated with DNase using the TurboDNase kit (Life Technologies). Complementary DNA was prepared using Superscript III reverse transcriptase (Life Technologies). Transcription was detected by qPCRs containing 500 nM of each primer (Additional file 2: Table S1), 50 μM dNTPs, 2 μM MgCl, 8 μM Syto9 green fluorescent stain (Life Technologies) and GoTaq DNA polymerase (Promega). Reactions were incubated through 40 cycles of 95 °C for 30 s, 60 °C for 30 s and 72 °C for 60 s. Relative levels of transcription of each ORF were calculated by comparing cycle threshold (Ct) values for each ORF to that of the host housekeeping gene GAPDH and to those obtained for uninfected cell controls, determining the normalised expression value as previously described [43, 44]. Further amino acid sequence analyses, as described above, were continued only on the polypeptides encoded by ORFs confirmed to be transcribed in vitro.

### Availability of supporting data

The MaHV-1 genome sequence data has been submitted to GenBank and the accession number is KT594769. The Illumina read data have been submitted to the Short Reads Archive database and has the ID number SRA:SRP067309.

## Additional files

Additional file 1: Figure S1. Relative transcript levels for the unique hypothetical MaHV-1 ORFs PW1 to PW6 at 4 h (grey bars) and 12 h (black bars) post infection (hpi) in wallaby fibroblast cells. Expression was normalised to the host housekeeping gene, GAPDH, and analysed by calculating mean normalised expression values. No viral transcripts were detected in uninfected cells. Error bars indicate standard deviation of three biological replicates. (TIF 8005 kb) Additional file 2: Table S1. Oligonucleotides used for ORF transcription studies of the unique hypothetical MaHV-1 ORFs (PW1 to PW6) and the host housekeeping gene GAPDH. (DOC 37 kb)

## Abbreviations

Ct: cycle threshold
DPOL: DNA polymerase
FbHV: fruit bat herpesvirus
GAPDH: Glyceraldehyde 3-phosphate dehydrogenase
gB: glycoprotein B
gD: glycoprotein D
gG: glycoprotein G
gJ: glycoprotein J
HHV: human herpesvirus
hpi: hours post infection
ICP: infected cell protein
IR/TR: internal repeat/ terminal repeat
I-TASSER: Iterative Threading ASSEmbly Refinement algorithm
MaHV: macropodid herpesvirus
ORF: open reading frame
oriLyt: origin of lytic replication
PDB: protein data bank
pfam: protein families database
PRV: pseudorabies virus / suid herpesvirus 1
PSSM: position-specific scoring matrix
PW: parma wallaby
qRT-PCR: quantitative reverse transcription polymerase chain reaction
RMSD: root-mean-square deviation
SRA: short reads archive
TCID: tissue culture infective dose
UL: unique long
US: unique short
VZV: varicella zoster virus

## References

[1] Finnie EP, Littlejohns IR, Acland HM (1976). Letter: Mortalities in parma wallabies (*Macropus parma*) associated with probable herpesvirus. Aust Vet J.

[2] Callinan RB, Kefford B (1981). Mortalities associated with herpesvirus infection in captive macropods. J Wildl Dis.

[3] Wilks CR, Kefford B, Callinan RB (1981). Herpesvirus as a cause of fatal disease in Australian wallabies. J Comp Pathol.

[4] Webber CE, Whalley JM (1978). Widespread occurrence in Australian marsupials of neutralizing antibodies to a herpesvirus from a parma wallaby. Aust J Exp Biol Med Sci.

[5] Kerr A, Whalley JM, Poole WE (1981). Herpesvirus neutralising antibody in grey kangaroos. Aust Vet J.

[6] Vaz PK, Motha J, McCowan C, Ficorilli N, Whiteley PL, Wilks CR (2013). Isolation and characterization of a novel herpesvirus from a free-ranging eastern grey kangaroo (*Macropus giganteus*). J Wildl Dis.

[7] Wilcox RS, Vaz P, Ficorilli NP, Whiteley PL, Wilks CR, Devlin JM (2011). Gammaherpesvirus infection in a free-ranging eastern grey kangaroo (*Macropus giganteus*). Aust Vet J.

[8] Smith JA, Wellehan JF, Pogranichniy RM, Childress AL, Landolfi JA, Terio KA (2008). Identification and isolation of a novel herpesvirus in a captive mob of eastern grey kangaroos (*Macropus giganteus*). Vet Microbiol.

[9] Stalder K, Vaz PK, Gilkerson JR, Baker R, Whiteley P, Ficorilli N (2015). Prevalence and clinical significance of herpesvirus infection in populations of Australian marsupials. PLoS One.

[10] Amery-Gale J, Vaz PK, Whiteley P, Tatarczuch L, Taggart DA, Charles JA (2014). Detection and identification of a gammaherpesvirus in Antechinus spp. in Australia. J Wildl Dis.

[11] Vaz P, Whiteley PL, Wilks CR, Browning GF, Gilkerson JR, Ficorilli N (2012). Detection of a second novel gammaherpesvirus in a free-ranging koala (*Phascolarctos cinereus*). J Wildl Dis.

[12] Vaz P, Whiteley PL, Wilks CR, Duignan PJ, Ficorilli NP, Gilkerson JR (2011). Detection of a novel gammaherpesvirus in koalas (*Phascolarctos cinereus*). J Wildl Dis.

[13] Portas T, Fletcher D, Spratt D, Reiss A, Holz P, Stalder K (2014). Health evaluation of free-ranging eastern bettongs (*Bettongia gaimardi*) during translocation for reintroduction in Australia. J Wildl Dis.

[14] Roizmann B, Desrosiers RC, Fleckenstein B, Lopez C, Minson AC, Studdert MJ (1992). The family Herpesviridae: an update. The Herpesvirus Study Group of the International Committee on Taxonomy of Viruses. Arch Virol.

[15] Johnson MA, Whalley JM (1990). Structure and physical map of the genome of parma wallaby herpesvirus. Virus Res.

[16] Johnson MA, Whalley JM, Littlejohns IR, Dickson J, Smith VW, Wilks CR (1985). Macropodid herpesviruses 1 and 2: two herpesviruses from Australian marsupials differentiated by restriction endonucleases, DNA composition and hybridization. Brief report. Arch Virol.

[17] Johnson MA, Whalley JM (1987). Restriction enzyme maps of the macropodid herpesvirus 2 genome. Arch Virol.

[18] Guliani S, Polkinghorne I, Smith GA, Young P, Mattick JS, Mahony TJ (2002). Macropodid herpesvirus 1 encodes genes for both thymidylate synthase and ICP34.5. Virus Genes.

[19] Mahony TJ, Smith GA, Thomson DM (1999). Macropodid herpesviruses 1 and 2 occupy unexpected molecular phylogenic positions within the *Alphaherpesvirinae*. J Gen Virol.

[20] Bininda-Emonds ORP, Cardillo M, Jones KE, MacPhee RDE, Beck RMD, Grenyer R (2007). The delayed rise of present-day mammals. Nature.

[21] McGeoch DJ, Cook S (1994). Molecular phylogeny of the alphaherpesvirinae subfamily and a proposed evolutionary timescale. J Mol Biol.

[22] McGeoch DJ, Cook S, Dolan A, Jamieson FE, Telford EA (1995). Molecular phylogeny and evolutionary timescale for the family of mammalian herpesviruses. J Mol Biol.

[23] Thomson DM: In vitro characterisation of Macropodid herpesvirus 1 as a vaccine vector. PhD Thesis. The University of Queensland. Queensland: Australia;2002. http://espace.library.uq.edu.au/view/UQ:105884.

[24] Baines JD, Roizman B (1991). The open reading frames UL3, UL4, UL10, and UL16 are dispensable for the replication of herpes simplex virus 1 in cell culture. J Virol.

[25] Jahedi S, Markovitz NS, Filatov F, Roizman B (1999). Colocalization of the herpes simplex virus 1 UL4 protein with infected cell protein 22 in small, dense nuclear structures formed prior to onset of DNA synthesis. J Virol.

[26] Markovitz NS, Roizman B (2000). Small dense nuclear bodies are the site of localization of herpes simplex virus 1 UL3 and UL4 proteins and of ICP22 only when the latter protein is present. J Virol.

[27] Xing J, Wang S, Lin F, Pan W, Hu CD, Zheng C (2011). Comprehensive characterization of interaction complexes of herpes simplex virus type 1 ICP22, UL3, UL4, and UL20.5. J Virol.

[28] Babra B, Watson G, Xu W, Jeffrey BM, Xu J-R, Rockey DD (2012). Analysis of the genome of leporid herpesvirus 4. Virology.

[29] Sasaki M, Setiyono A, Handharyani E, Kobayashi S, Rahmadani I, Taha S (2014). Isolation and characterization of a novel alphaherpesvirus in fruit bats. J Virol.

[30] Aubert M, Chen Z, Lang R, Dang CH, Fowler C, Sloan DD (2008). The antiapoptotic herpes simplex virus glycoprotein J localizes to multiple cellular organelles and induces reactive oxygen species formation. J Virol.

[31] Ushijima Y, Goshima F, Kimura H, Nishiyama Y (2009). Herpes simplex virus type 2 tegument protein UL56 relocalizes ubiquitin ligase Nedd4 and has a role in transport and/or release of virions. Virol J.

[32] Koshizuka T, Goshima F, Takakuwa H, Nozawa N, Daikoku T, Koiwai O (2002). Identification and characterization of the UL56 gene product of herpes simplex virus type 2. J Virol.

[33] Koshizuka T, Kawaguchi Y, Goshima F, Mori I, Nishiyama Y (2006). Association of two membrane proteins encoded by herpes simplex virus type 2, UL11 and UL56. Virus Genes.

[34] Berkowitz C, Moyal M, Rosen-Wolff A, Darai G, Becker Y (1994). Herpes simplex virus type 1 (HSV-1) UL56 gene is involved in viral intraperitoneal pathogenicity to immunocompetent mice. Arch Virol.

[35] Kehm R, Rosen-Wolff A, Darai G (1996). Restitution of the UL56 gene expression of HSV-1 HFEM led to restoration of virulent phenotype; deletion of the amino acids 217 to 234 of the UL56 protein abrogates the virulent phenotype. Virus Res.

[36] Lee S-W, Devlin JM, Markham JF, Noormohammadi AH, Browning GF, Ficorilli NP (2011). Comparative analysis of the complete genome sequences of two Australian origin live attenuated vaccines of infectious laryngotracheitis virus. Vaccine.

[37] Kearse M, Moir R, Wilson A, Stones-Havas S, Cheung M, Sturrock S (2012). Geneious Basic: an integrated and extendable desktop software platform for the organization and analysis of sequence data. Bioinformatics.

[38] Altschul SF, Gish W, Miller W, Myers EW, Lipman DJ (1990). Basic local alignment search tool. J Mol Biol.

[39] Boratyn GM, Camacho C, Cooper PS, Coulouris G, Fong A, Ma N (2013). BLAST: a more efficient report with usability improvements. Nucleic Acids Res.

[40] Zhang Y (2008). I-TASSER server for protein 3D structure prediction. BMC Bioinformatics.

[41] Jukes T, Cantor C, Munro M (1969). evolution of protein molecules. Mammalian protein metabolism.

[42] Uren J, Moore R, van den Brenk HA (1966). Development of a pseudodiploid cell line, JU56, of wallaby fibroblasts. Exp Cell Res.

[43] Muller PY, Janovjak H, Miserez AR, Dobbie Z (2002). Processing of gene expression data generated by quantitative real-time RT-PCR. Biotechniques.

[44] Muller PY, Janovjak H, Miserez AR, Dobbie Z (2002). Processing of gene expression data generated by quantitative real-time RT PCR (vol 32, pg 1378, 2002). Biotechniques.

