# Supplementary figures and images for: The first genome sequence of a metatherian herpesvirus: Macropodid herpesvirus 1
Source: BMC Genomics. 2016 Jan 22;17:70. doi: 10.1186/s12864-016-2390-2 (PMC4724163; doi:10.1186/s12864-016-2390-2)

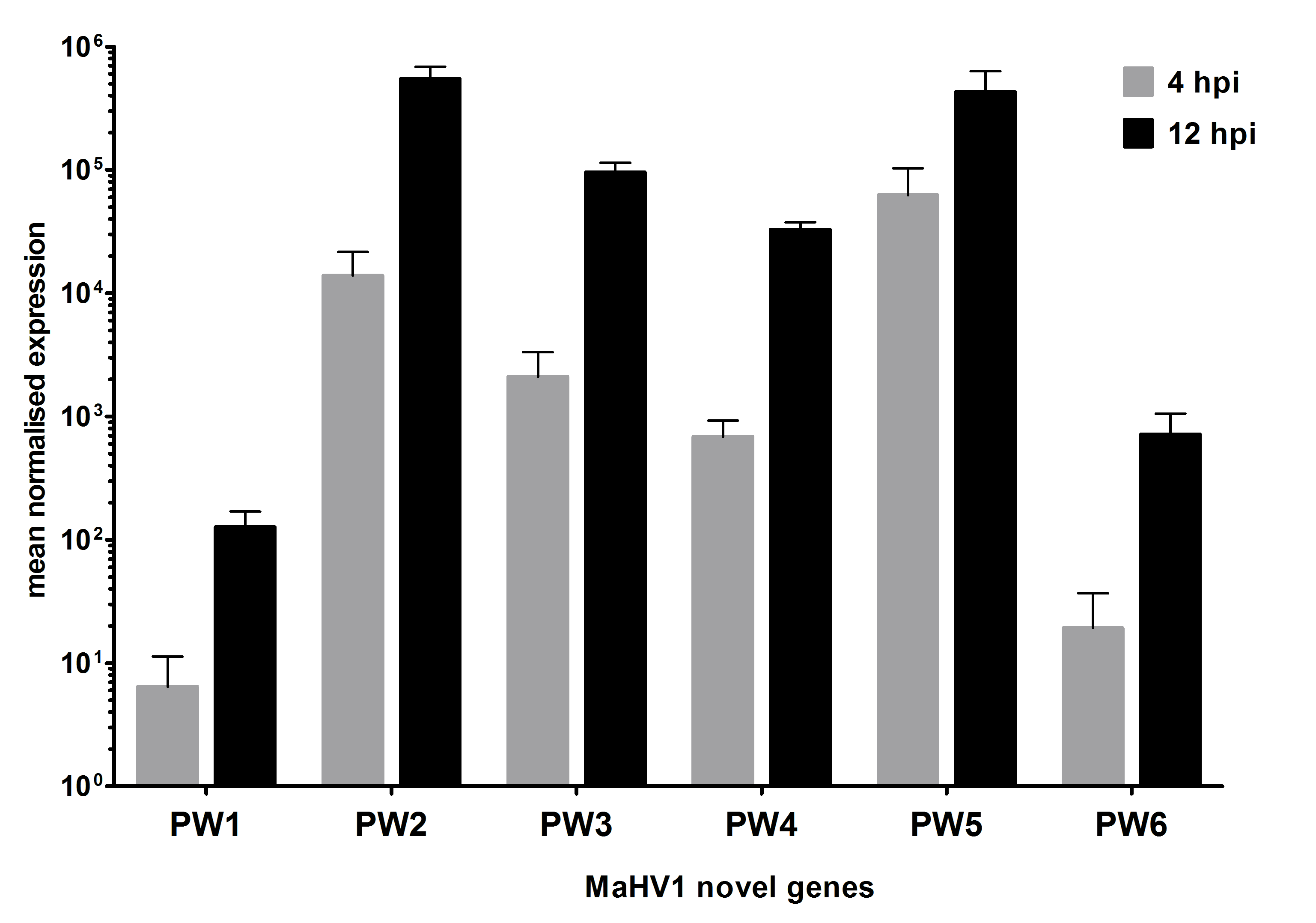

Supplement: Additional file 1: Figure S1. — Relative transcript levels for the unique hypothetical MaHV-1 ORFs PW1 to PW6 at 4 h (grey bars) and 12 h (black bars) post infection (hpi) in wallaby fibroblast cells. Expression was normalised to the host housekeeping gene, GAPDH, and analysed by calculating mean normalised expression values. No viral transcripts were detected in uninfected cells. Error bars indicate standard deviation of three biological replicates. (TIF 8005 kb) [file 12864_2016_2390_MOESM1_ESM.tif]
