# Supplementary material for: The first genome sequence of a metatherian herpesvirus: Macropodid herpesvirus 1
Source: BMC Genomics. 2016 Jan 22;17:70. doi: 10.1186/s12864-016-2390-2 (PMC4724163; doi:10.1186/s12864-016-2390-2)
Supplement: Additional file 2: Table S1. — Oligonucleotides used for ORF transcription studies of the unique hypothetical MaHV-1 ORFs (PW1 to PW6) and the host housekeeping gene GAPDH. (DOC 37 kb) [file 12864_2016_2390_MOESM2_ESM.doc]

**Supplementary Table 1**. Oligonucleotides used for ORF transcription studies of the unique hypothetical MaHV-1 ORFs (PW1 to PW6) and the host housekeeping gene GAPDH.

| **Organism** | **Target ORF** | **Primer name** | **Direction** | **5´ – 3´ Sequence** |
| --- | --- | --- | --- | --- |
| Host | GAPDH | MaGAPDH fwd | forward | GGACTCATGACCACAGT |
|  |  | MaGAPDH rev | reverse | CCATCACGCCACAGC |
| MaHV-1 | PW1 | PW1 fwd2 | forward | GGTAGGGTGGTCAGTAGAG |
|  |  | PW1 rev2 | reverse | CGTTCGAAGATGGGCGAGATT |
|  | PW2 | PW2 fwd2 | forward | GCCATAGAATAAGAGCGCCC |
|  |  | PW2 rev2 | reverse | CGCGTCCTACCGTACATTC |
|  | PW3 | PW3 fwd2 | forward | CTTTACGCCGAGACCTCTATTC |
|  |  | PW3 rev2 | reverse | GTAGTGCAGTTTGTCCAGCG |
|  | PW4 | PW4 fwd2 | forward | TCAGTTATGGAAAATTGCCCGC |
|  |  | PW4 rev2 | reverse | CCTTGGGAATTTAGAGGACTTG |
|  | PW5 | PW5 fwd2 | forward | GCTCTCCATTATGGAGCAAGCC |
|  |  | PW5 rev2 | reverse | TGGCTGTTGCTCTCTCGGTGTC |
|  | PW6 | PW6 fwd2 | forward | TATGTACCCGACAACCAGCC |
|  |  | PW6 rev2 | reverse | GAGAAACATGTAGTTGCGTGC |
